# *OsSUV3* transgenic rice maintains higher endogenous levels of plant hormones that mitigates adverse effects of salinity and sustains crop productivity

**DOI:** 10.1186/s12284-014-0017-2

**Published:** 2014-09-02

**Authors:** Ranjan Kumar Sahoo, Mohammad Wahid Ansari, Renu Tuteja, Narendra Tuteja

**Affiliations:** 1 Plant Molecular Biology Group, International Centre for Genetic Engineering and Biotechnology (ICGEB), Aruna Asaf Ali Marg, New Delhi 110 067, India

**Keywords:** Helicases, Oryza sativa, Plant hormones, Plant tolerance, Salinity stress

## Abstract

**Background:**

The *SUV3* (suppressor of Var 3) gene encodes a DNA and RNA helicase, which is localized in the mitochondria. Plant SUV3 has not yet been characterized in detail. However, the *Arabidopsis* ortholog of SUV3 (AT4G14790) has been shown to be involved in embryo sac development. Previously, we have reported that rice SUV3 functions as DNA and RNA helicase and provides salinity stress tolerance by maintaining photosynthesis and antioxidant machinery. Here, we report further analysis of the transgenic *OsSUV3* rice plants under salt stress.

**Findings:**

The transgenic OsSUV3 overexpressing rice T_1_ lines showed significantly higher endogenous content of plant hormones *viz.,* gibberellic acid (GA_3_), zeatin (Z) and indole-3-acetic acid (IAA) in leaf, stem and root as compared to wild-type (WT), vector control (VC) and antisense (AS) plants under salt (200 mM NaCl) stress condition. A similar trend of endogenous plant hormones profile was also reflected in the T_2_ generation of *OsSUV3* transgenic rice under defined parameters and stress condition.

**Conclusions:**

In response to stress, OsSUV3 rice plants maintained plant hormone levels that regulate the expression of several stress-induced genes and reduce adverse effects of salt on plant growth and development and therefore sustains crop productivity.

## Findings

Abiotic stresses (such as high salinity, drought, flood and high and low temperatures) cause the greatest constraint on crop productivity. These affect the growth and productivity and trigger a series of biochemical and molecular changes in the plants, which appear in the form of morphological and physiological variations in the crops. Globally, it has been estimated that approximately 70% of yield reduction is the direct result of the negative effects of abiotic stresses on various crops (Acquaah [2007]). High salinity causes the hyper-ionic and hyper-osmotic stress to plant cells leading to reduction in plant growth and productivity, which in severe cases may lead to plant death. Plants may cope with the adverse effects of high salinity by improving their photosynthesis and antioxidant machinery (Tuteja [2007]; Tuteja et al. [2013]). The rice SUV3 helicase has been shown to be involved in salinity, cadmium and zinc stress tolerance (Tuteja et al. [2013]; Sahoo and Tuteja [2014]). The endogenous content of plant hormones directs the molecular and biochemical mechanisms to confer increased stress tolerance to improve plant growth and development and thereby better survival (Osakabe et al. [2013]).

Plant hormones regulate several aspects of plant growth and developmental processes in response to the multiple abiotic and biotic stresses. The molecular events in plant hormone responses and environmental stress adjustment have been reported (Harrison [2012]). Gibberellic acid (GA_3_) has been reported to be involved in plant response to abiotic stress and GA_3_ mediated growth variability in plants relieves the adverse effects of salt, oxidative, and heat stresses (Qin et al. [2011]). Stress-induced production of cytokinin in plants facilitates sink strengthening via a cytokinin-dependent co-ordinated regulation of carbon and nitrogen metabolism that provides an improved tolerance to salinity and drought stress (Ha et al. [2012]). In response to stress, plants synchronize their development by exogenous signals and signal transduction cascades. The essential roles of auxin in various growth and developmental processes have been well documented. Additionally, the role of auxin in the regulation of biotic and abiotic stresses has been suggested (Kazan et al. [2013]).

The *OsSUV3* gene (1.74 kb) (accession number: GQ982584; locus ID NM_001057785 on chromosome 3) was cloned previously (Tuteja et al. [2013]). The OsSUV3 gene shows high homology with previously existing sequences (accession numbers AK101069.1; XM_006650512.1) in the GenBank (http://blast.ncbi.nlm.nih.gov/Blast.cgi). The IR64 rice transgenic plants overexpressing *OsSUV3* in sense and antisense orientations were developed as described previously (Tuteja et al. [2013]). A competent strain of *Agrobacterium tumefacien*s (LBA4404) transformed with empty vector (pCAMBIA1301) construct was used as vector control to raise control plants of rice via standard protocol as described earlier (Tuteja et al. [2013]). The plants *viz.,* T_1_*OsSUV3* rice, T_2_*OsSUV3* rice, wild-type (WT), vector control (VC), and antisense (AS) were allowed to grow under 200 mM salt (NaCl). After 24 h, the plant samples such as leaves, stem and root were collected for extraction and purification of the endogenous plant hormones (GA_3_, zeatin and IAA) by adopting method of Chen et al. ([1996]).

**Figure 1:**
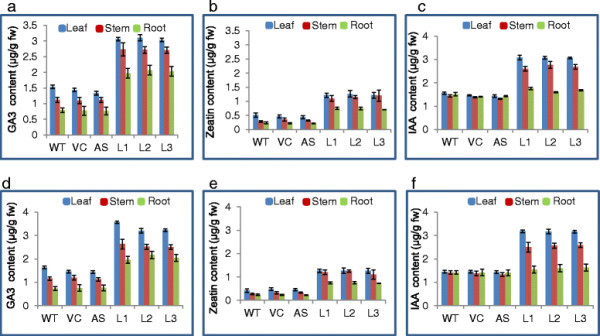
**Plant hormones gibberellic acid (GA**_**3**_**), zeatin and indole-3-acetic acid (IAA) profile in the transgenic** ***OsSUV3*** **rice plants under salt stress.** Endogenous content (μg/g fw) of GA_3_, zeatin and IAA in *OsSUV3* transgenic rice lines (L1, L2 and L3) of T_1_ generation **(a-c)** and T_2_ generation **(d-f)** under 200 mM NaCl. The significant difference between the mean values (n = 3) of rice plants (WT, VC and AS) and *OsSUV3* overexpressing transgenic rice lines (L1-L3) was determined by one-way analysis of variance (ANOVA) using SPSS 10.0 (SPSS, Inc., now IBM, http://www-01.ibm.com/software/analytics/spss). The WT, VC and transgenic lines at P < 0.05, P < 0.01 and P < 0.001 were considered statistically significant.

The *OsSUV3* transgenic rice lines *viz.,* Line 1 (L1), Line 2 (L2) and Line 3 (L3) showed higher endogenous content of plant hormones as compared to AS, VC and WT plants (Figure 1a-f). The plant hormones profile of transgenic *OsSUV3* rice was assessed upto T_2_ generation. In T_1_ generation, GA_3_ content in leaves of *OsSUV3* transgenic rice lines (L1, L2 and L3) was relatively higher (3.07, 3.1 and 3.04 μg/g fw, respectively) as compared to WT (1.54 μg/g fw), VC (1.44 μg/g fw) and AS (1.34 μg/g fw). GA_3_ content was 2.7, 2.72 and 2.7 μg/g fw in shoots and 1.9, 2.1 and 2.0 μg/g fw in roots of *OsSUV3* rice lines (Figure 1a). Zeatin ranged between 0.7-1.27 μg/g fw, whereas control plants showed 0.2-0.52 of zeatin (μg/g fw) in leaf, shoot and roots (Figure 1b). The endogenous level of IAA in leaf, shoot and roots of transgenic rice lines ranged between 1.6-3.1 μg/g fw as opposed to control plants (1.4-1.6 μg/g fw) (Figure 1c). A similar trend of phytohormones (GA_3_, zeatin and IAA) was also reflected in T_2_ plants of *OsSUV3* transgenic rice lines (L1, L2 and L3) (Figure 1d-f).

Multiple reports of effects of salinity on plants are available (Mahajan and Tuteja [2005]; Zolla et al. [2010]). To overcome the salinity stress-induced loss of crop productivity, investigations have focused more on the mechanisms of salt tolerance in plants (Tuteja [2007]). We recently reported that *OsSUV3* over expression in rice functions in salt tolerance, it also improved growth performance in terms of plant height, number of tillers/plant, number of panicle/plant, number of filled grain/panicle, number of non-chaffy grains/panicle, straw dry weight, 100 grain weight, root length, root dry weight, leaf area, root and shoot lengths when compared to control plants (Tuteja et al. [2013]). Much attention is now focused on the plant hormones for their crucial roles in stress responses and adaptation (Kuppu et al. [2013]) and exploitation of different plant hormones for reducing the negative effects of salinity in growth parameters (Egamberdieva [2009]). In the present study, *OsSUV3* rice transgenic T_1_ and T_2_ lines maintained higher endogenous content of GA_3_, zeatin and IAA under 200 mM NaCl as compared to the control plants. The findings of the present investigation suggest that plant hormones mitigate various adverse effects of salt to sustain plant productivity via modulating metabolism and signalling events in plants.

## Competing interests

The authors declare no potential competing interests.

## Authors’ contributions

NT conceived and designed the experiments. RKS screened the *OsSUV3* rice transgenic lines and performed the experiments. MWA and RT performed data analysis, interpreted the results, discussed the data and drafted the manuscript. All authors have read and approved the final manuscript.
